# Oil Hydrocarbon Degradation by Caspian Sea Microbial Communities

**DOI:** 10.3389/fmicb.2019.00995

**Published:** 2019-05-09

**Authors:** John I. Miller, Stephen Techtmann, Julian Fortney, Nagissa Mahmoudi, Dominique Joyner, Jiang Liu, Scott Olesen, Eric Alm, Adolfo Fernandez, Piero Gardinali, Nargiz GaraJayeva, Faig S. Askerov, Terry C. Hazen

**Affiliations:** ^1^Department of Civil and Environmental Engineering, The University of Tennessee, Knoxville, Knoxville, TN, United States; ^2^Oak Ridge National Laboratory, Oak Ridge, TN, United States; ^3^Biosciences Division, Michigan Technological University, Houghton, MI, United States; ^4^Department of Earth System Science, Stanford University, Stanford, CA, United States; ^5^Department of Earth and Planetary Sciences, McGill University, Montreal, QC, Canada; ^6^Harvard School of Public Health, Cambridge, MA, United States; ^7^Massachusetts Institute of Technology, Cambridge, MA, United States; ^8^Department of Chemistry and Biochemistry, Florida International University, Miami, FL, United States; ^9^BP, Baku, Azerbaijan

**Keywords:** 16S rRNA gene sequencing, Caspian Sea, anaerobic, cold temperature, marine microbial community, microbial ecology, oil biodegradation, petroleum

## Abstract

The Caspian Sea, which is the largest landlocked body of water on the planet, receives substantial annual hydrocarbon input from anthropogenic sources (e.g., industry, agriculture, oil exploration, and extraction) and natural sources (e.g., mud volcanoes and oil seeps). The Caspian Sea also receives substantial amounts of runoff from agricultural and municipal sources, containing nutrients that have caused eutrophication and subsequent hypoxia in the deep, cold waters. The effect of decreasing oxygen saturation and cold temperatures on oil hydrocarbon biodegradation by a microbial community is not well characterized. The purpose of this study was to investigate the effect of oxic and anoxic conditions on oil hydrocarbon biodegradation at cold temperatures by microbial communities derived from the Caspian Sea. Water samples were collected from the Caspian Sea for study in experimental microcosms. Major taxonomic orders observed in the ambient water samples included *Flavobacteriales*, *Actinomycetales*, *and Oceanospirillales*. Microcosms were inoculated with microbial communities from the deepest waters and amended with oil hydrocarbons for 17 days. Hydrocarbon degradation and shifts in microbial community structure were measured. Surprisingly, oil hydrocarbon biodegradation under anoxic conditions exceeded that under oxic conditions; this was particularly evident in the degradation of aromatic hydrocarbons. Important microbial taxa associated with the anoxic microcosms included known oil degraders such as *Oceanospirillaceae*. This study provides knowledge about the ambient community structure of the Caspian Sea, which serves as an important reference point for future studies. Furthermore, this may be the first report in which anaerobic biodegradation of oil hydrocarbons exceeds aerobic biodegradation.

## Introduction

The Caspian Sea is the largest enclosed body of water on earth, with a volume of 78,000 km^3^ and a surface area of 3.8 × 10^5^ km^2^ (Dumont, 1998; Tuzhilkin et al., 2005) and has been landlocked for five million years. Its primary water inputs are from river runoff, with more than 130 freshwater river inputs, and precipitation, causing the waters of the Caspian of today to be brackish, with salinity values approximately one third of ocean seawater (Leroy et al., 2007). Geographically the sea is divided into three basins: north, central, and south.

The southern basin of the Caspian Sea is the deepest of the three basins in the Caspian (1025 m). The surface waters are warm (∼25°C) but fluctuate seasonally. There is a thermocline around 15–40 m, and temperatures in the deepest waters (>100 m) are constant year-round at 6.8°C (Tuzhilkin et al., 2005). Freshwater input from rivers is nutrient-rich, deriving from industrial and agricultural runoff, and therefore contributes substantial pollution to the Caspian Sea, and this has increased in recent years (Zonn, 2005). This causes eutrophication, resulting in hypoxia in the deep-waters (Diaz, 2001). Since the middle of the 20th century, the deep-waters have declined from ∼26% oxygen saturation to ∼5% (Chicherina et al., 2004; Tuzhilkin et al., 2005). Hypoxic zones may have negative consequences such as increased mortality of benthic organisms, decreased biodiversity, and altered biochemical cycles (Diaz and Rosenberg, 1995). Furthermore, oil and gas exploration and production from the Caspian Sea have also increased in recent years. Petroleum hydrocarbon inputs are estimated to be between 70 and 90 tons per year (Chicherina et al., 2004), with total petroleum hydrocarbon concentrations ranging from 0.12 mg/L in the shallow water to <0.02 mg/L in the deep water (Korshenko and Gul, 2005). The highest petroleum hydrocarbon concentrations have been reported in the southern basin and in surface waters and sediments, but are “almost undetectable” in the water column below 500 m (Korshenko and Gul, 2005). The unique combination of physical and chemical variables may have caused adaptations within the microbial community of the deep waters of the Caspian Sea.

Biodegradation of contaminants by microbial communities is an important process. Many contaminants, especially organic compounds (e.g., oil hydrocarbons) are derived from natural compounds or have natural analogs in the environment (Brakstad et al., 2014) Indigenous microbial taxa and communities may adapt to contaminants (e.g., via natural selection) if the exposure continues for an extended or short period of time (Brakstad et al., 2014; Smith et al., 2015). Additionally, opportunistic organisms are known to become enriched in the event of an influx of hydrocarbons into a system (Bælum et al., 2012; Hazen et al., 2016), but vary in their relative abundance and contaminant degrading abilities (Bælum et al., 2012). To date, there are few studies on the microbial community structure and function in the Caspian Sea, despite extensive long-term geochemical and hydrological studies. The research that is available about the microbial community of the Caspian Sea is mostly high level, general knowledge and tends to be similar to other marine environments (Salmanov, 2006). The upper 100 m of the water column is dominated by aerobic strains and high microbial cell counts (∼6 × 10^5^ cells/mL). The deeper waters, on the other hand, have lower microbial cell counts (∼9 × 10^4^ cells/mL), and the benthic layers are dominated by anaerobic strains.

Hydrocarbon degrading *Bacteria* and fungi have been isolated from the coastal waters and sediments of the Caspian Sea (Vasheghani-Farahani and Mehrnia, 2000; Shkidchenko and Arinbasarov, 2002; Salmanov, 2006; Veliev et al., 2008; Hassanshahian et al., 2010, 2012; Kulikova et al., 2010; Safary et al., 2010; Mahmoudi et al., 2015), but the taxonomic identity of these isolates was often uninvestigated. Several of the identified isolates (e.g., *Pseudomonas* sp. and *Gordonia* sp.) demonstrated growth on crude oil (Hassanshahian et al., 2012). Most probable number experiments indicate that heterotrophic *Bacteria* in coastal sediments are high (2.5 × 10^5^ cells/mL), and that hydrocarbon degrading *Bacteria* are also high (Hassanshahian et al., 2010). It is evident that many oil degrading microbes exist in the shallow waters of the Caspian Sea, but the structure of the community has not been investigated. Furthermore, the microbial community of the deep waters, which are hypoxic, has not been investigated.

Anaerobic biodegradation of hydrocarbons is a significant process that occurs in many environments (Wawrik et al., 2012; Gründger et al., 2015; Gieg and Toth, 2016; Laso-Pérez et al., 2016) and may be an important process in the deep-water communities of the Caspian Sea. Anaerobic hydrocarbon degradation is most frequently reported to be slower than aerobic hydrocarbon degradation (Widdel et al., 2010), with biodegradation coefficients of 0.445/d and 0.522/d, respectively (Suarez and Rifai, 1999). Anaerobic hydrocarbon degradation has been studied in a variety of environments and conditions including the following: fossil hydrocarbon reserves (Berdugo-Clavijo and Gieg, 2014), thermophilic communities (Laso-Pérez et al., 2016), and in microcosms inoculated with sediment under sulfate-reducing conditions (Sherry et al., 2013). However, less knowledge exists about this process in psychrophilic communities like those from the deep waters of the Caspian Sea.

The deep waters of the Caspian Sea represent a unique environment that is both cold and hypoxic and has a large influx of oil hydrocarbons. The impact of these conditions on the community structure has not been investigated. Previous investigations of this community most frequently characterized isolates from shallow, coastal waters, with fewer reports on the deep, oxygen deficient waters (Diarov and Serikov, 2006). The hypoxic conditions of the deep waters are likely to significantly affect the structures of these microbial communities and their mechanisms for hydrocarbon biodegradation. Understanding the process of anaerobic hydrocarbon degradation at cold temperatures by these deep-water communities has implications for potential bioremediation of this hydrocarbon contaminated marine environment and represents a unique ecological niche that, to date, has not been investigated.

## Materials and Methods

### Sample Collection and Environmental Variables

Water samples were collected from six sites in the southern basin of the Caspian Sea between July 27, 2013 and August 1, 2013. Sampling was conducted as part of BP’s oceanographic survey by Fugro, aboard the MV Svetlomor II. Niskin bottles were deployed for water collection. A MIDAS CTD + Profiler (Valeport Ltd, St. Peter’s Quay, United Kingdom) was attached to the sampling rosette for continuous monitoring of physical and chemical water variables. Environmental variables (temperature, dissolved oxygen, salinity, pH, turbidity) were measured continually through the water column (Figure 2).

Sample sites were chosen to sample diverse sea floor features (Table 2), both natural and man-made (sites 2–6), and one control site with no known man-made features (site 1). Man-made features include drill cuttings splay, debris, and oil and gas wells (active and abandoned). Water samples were collected from 2 to 4 depths at each sample site, depending on the depth to seafloor. Sample depths for each site were selected to approximate the following categories (where appropriate): near surface, one-third depth from surface, two-thirds depth from surface, and near bottom. In total, nineteen samples were collected.

*In situ* sampling of ambient seawater was conducted as follows. Between 62 and 123 liters of ambient seawater were filtered using a large volume pump (McLane Research Laboratories, East Falmouth, MA, United States). Different amounts of water were sampled due to the differences in the amount of particulate matter at different sample locations, which affected filtration. The water was filtered through a 142 mm nylon membrane with a pore size of 0.2 μm (Sterlitech, Kent, WA, United States) and then stored at −20°C. One third of the filter was used for DNA analysis reported here.

Immediately following string recovery, seawater was dispensed into 4-liter amber bottles using clean TYGON Tubing (Saint-Gobain, La Défense, Courbevoie, France) to limit aeration. Bottles were stored on-ship at 4°C and then shipped on wet ice. Forty mL of water was fixed in 4% formaldehyde and stored at 4°C for acridine orange direct counts (AODC)s.

One hundred mL of water was frozen at −20°C for analysis of dissolved organic carbon and nutrients. Total organic carbon and total nitrogen were analyzed with TOC-L analyzer (Shimadzu Scientific Instruments, Columbia, MD, United States), and inorganic nutrients were analyzed with a SEAL AutoAnalyzer 3 HR (SEAL Analytical Inc., Mequon, WI, United States). Nutrients (nitrate, nitrite, ammonia, total nitrogen, inorganic phosphate, silicate) were measured at each of the sampling sites and depths.

### Acridine Orange Direct Cell Counts

Acridine orange direct cell counts (AODC) were performed as describe by Francisco et al. (1973). Cell counts were performed with Zeiss Axioskop epifluorescence microscope (Carl Zeiss, Inc., Germany).

### Microcosms Experiments

Laboratory microcosm experiments were prepared to investigate (1) the change in microbial community composition and structure and (2) its potential for oil biodegradation under oxic and anoxic conditions. Microcosms were prepared in triplicate at 6°C using the deepest waters from stations (575 m) with either nitrogen (anoxic) or atmospheric (oxic) headspace. They were amended with either 100 ppm native oil hydrocarbons (E10 slot crude oil) or 100 ppm oil and 1 ppm Corexit dispersant (Nalco, Sugar Land, TX, United States) for comparison with Bælum et al. (2012). Control microcosms were killed and then amended as above. Carbon dioxide evolution was measured continually with a Micro-Oxymax Respirometer (Columbus Instruments International, Columbus, OH, United States) for 17 days. Microcosms were removed from the experiment in triplicate and killed at days 0, 3, and 17. One microcosm was used for 16S rRNA gene sequencing and two were used for hydrocarbon quantification.

### Hydrocarbon Analysis

Native oil hydrocarbon analog E10 slot (crude oil) was provided by BP. At 15°C, the API gravity of E10 slot oil was approximately 33°, and the density was 0.86 g/L. Samples from each microcosm at every timeline were sent to Florida International University and processed by liquid-liquid extraction with methylene chloride for hydrocarbon quantification. Saturated hydrocarbons and an estimation of total petroleum hydrocarbons (TPHs) were performed by gas chromatography-flame ionization detector (GC-FID) using a modification of SW-846 Method 8015. A subset of relevant polycyclic aromatic hydrocarbons (PAHs) including the 16 EPA priority PAHs and their alkylated homologues were analyzed using gas chromatography-mass spectrometry (GC/MS) in selected ion monitoring (SIM). The recalcitrant biomarker C30-hopane was also measured in the GC/MS method. This protocol is described in EARL-SOP-2000-O-109 and is based on a modification of previously reported procedures expanded to accommodate additional analytes (Denoux et al., 1998).

### DNA Extraction, 16S rRNA Gene Amplicon Sequencing, and Data Pre-preprocessing

Genomic DNA was extracted using methodology described by Miller et al. (1999) with modifications as described by Hazen et al. (2010). Then, genomic DNA was cleaned using the Genomic DNA Clean & Concentrator (DCC) kit (Zymo Research, Irvine, CA, United States). Quality of extracted DNA was determined by measuring the 260/280 and 260/230 ratios on a NanoDrop spectrophotometer (Thermo Fisher Scientific, Waltham, MA, United States). DNA concentration was determined by PicoGreen (Thermo Fisher Scientific, Waltham, MA, United States).

The 16S rRNA gene libraries were prepared as previously described (Caporaso et al., 2012). The V4 region of the 16S rRNA gene was amplified by PCR using universal primers 515f and barcoded 806r (which anneal to both *Bacterial* and *Archaeal* sequences) with Phusion DNA polymerase (Thermo Fisher Scientific, Waltham, MA, United States). A 12 base pair barcode index was included in the reverse primer to multiplex samples for sequencing analysis. The 16S rRNA gene amplicons were then pooled together, and the quality and size of the amplicons was analyzed using a Bioanalyzer (Agilent Technologies, Santa Clara, CA, United States). The 16S rRNA gene libraries were sequenced using a MiSeq with a V2 kit (Illumina, San Diego, CA, United States).

### Bioinformatics Processing of Sequence Data

The resulting sequence information was pre-processed with the QIIME wrapper software (Caporaso et al., 2010) on Biolinux (Field et al., 2006). Paired-end reads were joined using fastq-join (Aronesty, 2013), and then paired sequences were demultiplexed and quality filtered to remove reads with phred scores below 20 (using the QIIME script split_libraries_fastq.py -q 19). Chimera detection was then performed on demultiplexed, quality-filtered reads using UCHIME (Edgar, 2010; Edgar et al., 2011) including de novo and reference-based chimera detection. Chimera-checked sequences were clustered into OTUs at 97% sequence similarity using UCLUST (Edgar, 2010) and the open-reference clustering protocol with the GreenGenes 16S rRNA gene sequence database as reference (DeSantis et al., 2006). Counts of the reads comprising each OTU were exported to biom format (McDonald et al., 2012) for statistical analysis. Read counts were rarefied to a number of reads equal to that of the smallest sample. Then, OTUs that comprised <0.005% of total reads across all samples were removed from the data before downstream analysis.

Illumina MiSeq analysis of 16S rRNA gene amplicon sequences from the microcosms resulted in mean 389,492 (±477,528) reads per sample (after filtering). A total of 14,913 OTUs were identified, with mean 6,892 (±5,197) OTUs per sample. The raw sequence data were deposited in the sequence read archive of NCBI under accession number SRR8591396.

### Statistical Analysis

Samples were divided into two water masses (shallow and deep) for analysis of environmental variables and microbial communities; shallow waters were defined as waters ≤50 m in depth, and deep waters were defined as waters >50 m in depth, which is consistent with the observed thermocline (Figure 2). To test the hypothesis that environmental variables were different in the two water masses, the means of each parameter were compared by *t*-test for independent (unpaired) samples. For these and all following statistical tests, *P*-values were corrected for multiple comparisons using the Bonferroni method with the Statsmodels package (v0.8.0) in Python (v3.6) and considered significant if alpha <0.05.

The number of DNA reads in each sample was rarefied to 31,103 (which is the number of reads in the smallest sample) using the Scikit-Bio package (v0.4.2) in Python. Alpha-diversity was calculated using Shannon and Simpson diversity metrics, and richness was estimated using the chao1 metric, also with Scikit-Bio. ANOVA was used to test the hypothesis that alpha-diversity was different between water masses using the StatsModels package. Beta-diversity was calculated using weighted Unifrac in QIIME and the resulting distance matrix was used for non-metric multidimensional scaling (NMDS) with the vegan package (Oksanen et al., 2018) in R to explore the hypothesis that environmental variables influence the community structure. Environmental variables were fit to the ordination result also using the vegan package. Fisher’s exact test was applied to rarefied read counts using the SciPy package (v0.19.1) in Python to test the hypothesis that OTUs were enriched in either the shallow or deep-water samples. To determine which organisms responded to oil amendment during long-term exposure in microcosm experiments, the OTU read counts were transformed using the TEXMEX method (Olesen et al., 2016) in R. Following transformation, the change in community structure of the oil amended microcosms was compared to that of the un-amended microcosms in each atmospheric condition.

A three-way ANOVA was used to test for significant differences in hydrocarbon degradation between microcosm experiments with StatsModels. Significant factors were then used for *post hoc* tests (pairwise *t*-tests for independent samples).

## Results

### Site Characterization

**FIGURE 1:**
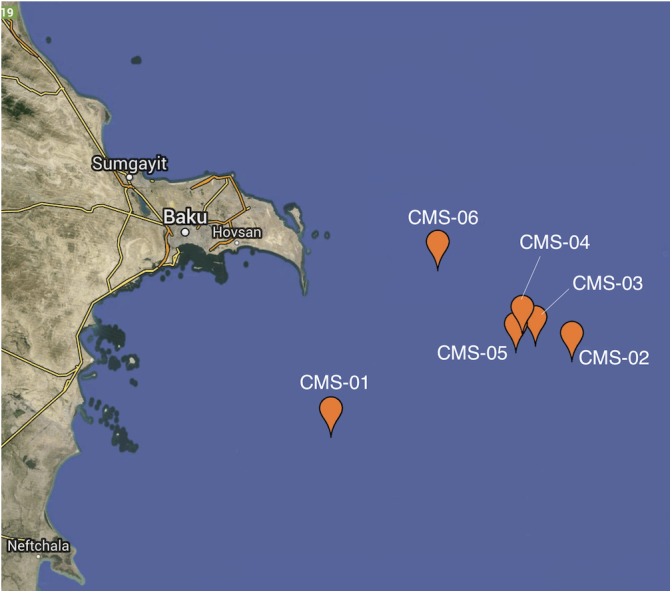
Map of sampling locations. Sample locations are in the Southern Basin of the Caspian Sea, off the coast of Azerbaijan. Each of the six sampling sites is indicated by a pin head.

**FIGURE 2:**
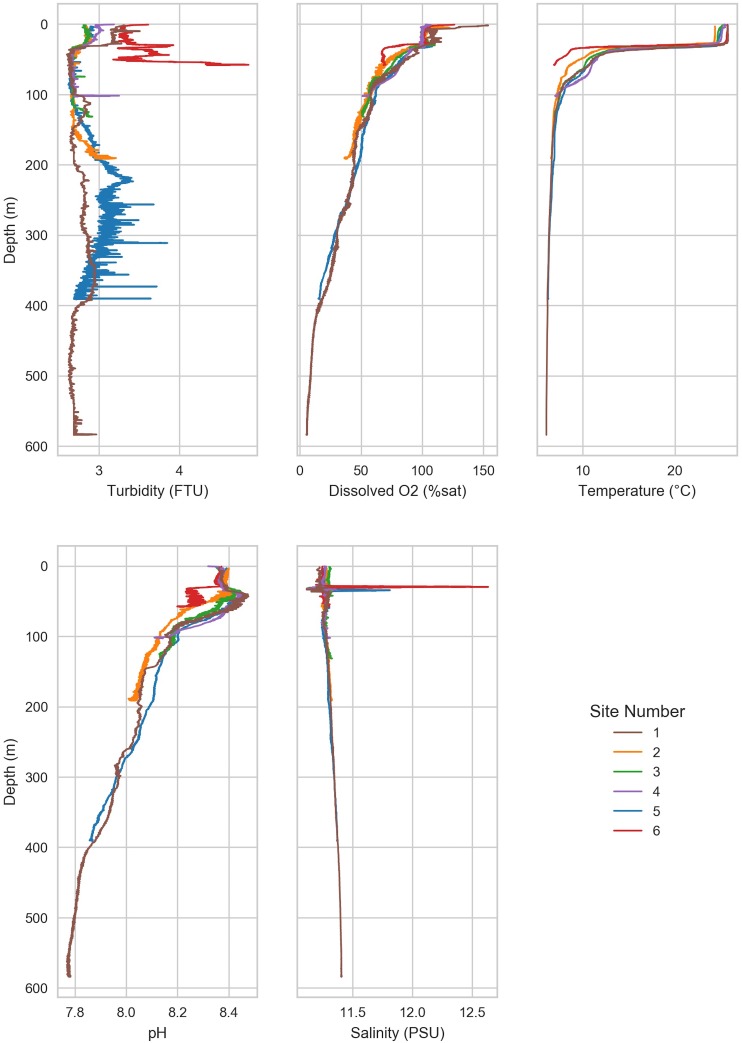
Environmental factors varied with depth. Environmental variables were measured throughout the water column from the surface water to near sea floor. A thermocline was observed at ∼50 m below sea level. Below the thermocline, dissolved oxygen and pH declined with depth.

Water samples were gathered from the Caspian Sea at 6 different sites (Figure 1). Depth to sea floor varied across the sites (Supplementary Table 1 and Figure 2). However, measurements of physical and chemical variables changed with depth in a similar manner at each of the 6 sites. Site 1 was the deepest (∼600 m), and site 6 was the shallowest (∼75 m).

A thermocline was observed at 50–100 m at all sites (Figure 2). The shallow waters (≤50 m) were warm (20.2 ± 5.1°C), but temperature declined rapidly and finally stabilized below 100 m (7.2 ± 1.4°C). The most rapid decline in temperature was observed at site 6, which dropped to 7.0°C at 58 m. The lowest observed temperature was 6.1°C in the deepest waters (Site 1, 575 m).

Dissolved oxygen (DO) in shallow waters was near saturation (8.38 ± 0.17 mg/L) but declined rapidly until ∼50 m (Figure 2 and Table 2). Below 50 m, DO declined slowly with depth in a similar manner across all sites, reaching 5 mg/L at ∼100 m, and continued to decline to <3 mg/L, which is the approximate upper limit for hypoxia (Hofmann et al., 2011), below 250 m. The lowest oxygen concentration (0.5 mg/L) was observed in the deep waters of site 1, which is considered severe hypoxia (Hofmann et al., 2011). Turbidity was constant with depth at most sites (2.9 ± 0.4 FTU). Turbidity was highest at site 6 (3.3–4.4 FTU), which is the shallowest site. Mean salinity was 11.3 ± 0.06 PSU, which is approximately one-third of mean ocean salinity (Wilson, 1975). The mean pH was 8.2 ± 0.2 and declined with depth from 8.4 (site 3) in the shallow waters to 7.8 in the deepest waters (site 1). All sites except for site 6 increased in pH slightly around 50 m before declining steadily with depth. Carbon, nitrogen, and phosphorous were higher than reported in other marine basins (Mahmoudi et al., 2015). The N:P ratio (63:1) for the sample collected from site 4 at 60 m depth was identified as an outlier and therefore removed from the following calculations (McGill et al., 1978). Ratio of N:P was near the Redfield ratio (12.1 ± 6.9) but varied substantially (range 2.30–25.0; Supplementary Table 2).

### *In situ* Microbial Community Characterization

Acridine orange direct counts ranged from 4.6 × 10^3^ to 2.3 × 10^5^ cells/ml across all samples (Supplementary Figure 1). The shallowest sites (3, 4, and 6) had the greatest range of AODCs throughout the water column. Although AODCs generally declined with depth across all sites, they varied little at site 1 throughout the water column (site 1 is the deepest site and the farthest from known man-made structures, Table 1).

**Table 1:** The important seafloor features near each sampling site are listed.

	Site number					
Feature	1	2	3	4	5	6
Drill cuttings splay (M)		Y	Y	Y	Y	
Embayment bounding ridge (N)				Y		
Eroded seabed (A)			Y			
Exposed landslide deposit (N)				Y		
Landslide visible at seabed (N)			Y			
Main landslide headwall scarp (N)			Y	Y		
Main landslide shear (N)			Y		Y	
Major crustal fault at seabed (N)				Y		
Man-made objects, debris (M)			Y	Y	Y	
Mud mound or mud volcano flow track (N)			Y		Y	
Mud volcano (N)	Y		Y	Y		
Plugged and abandoned oil and gas well (M)			Y	Y	Y	
Plugged and abandoned oil well (M)			Y	Y	Y	
Prospecting (M)			Y	Y	Y	
Tension crack (A)			Y			

*Sample sites were chosen in part based on important sea floor features. Some features are man-made (M), while others result unintentionally from anthropogenic activities (A). All other features are assumed to be natural (N).*

**FIGURE 3:**
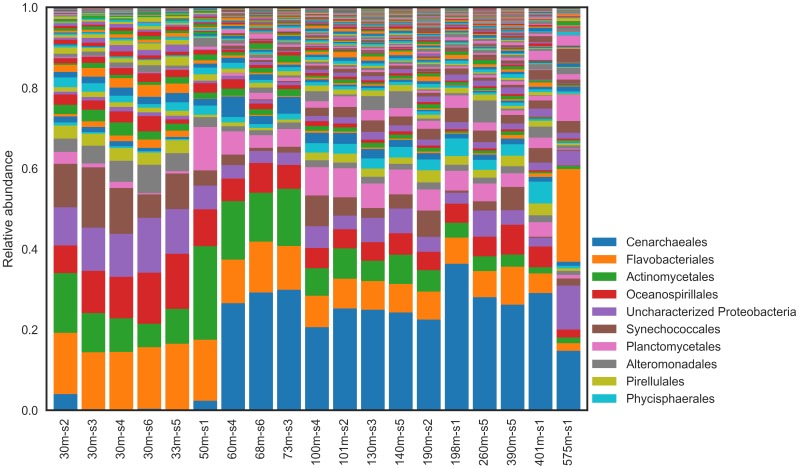
Relative abundance of microbial taxa in natural communities at Order level.

Microbial community composition and structure was investigated using 16S rRNA gene amplicon sequencing. Forty-three different phyla were identified across all sites and depths (from both *Bacteria* and *Archaea*). Several phyla were common to all samples and comprised a substantial fraction of the community (>5%; Figure 3). These included *Planctomycetes* (9.9 ± 3.5% of all reads), *Actinobacteria* (9.8 ± 5.8%), and *Verrucomicrobia* (5.1 ± 2.7%). Interestingly, unclassified *Proteobacteria* reads comprised a substantial fraction (10.3% ± 2.2) across all samples. Several orders of *Bacteria* were also important across all samples. These included *Actinomycetales* (8.3 ± 5.5%), *Oceanospirillales* (6.8 ± 2.9%), *Synechococcales* (5.3 ± 4.5%), and *Planctomycetales* (4.1 ± 2.9%). Neither alpha-diversity nor richness metrics were significantly different between shallow (≤50 m) and deep (>50 m) water communities (Supplementary Tables 3, 4).

**FIGURE 4:**
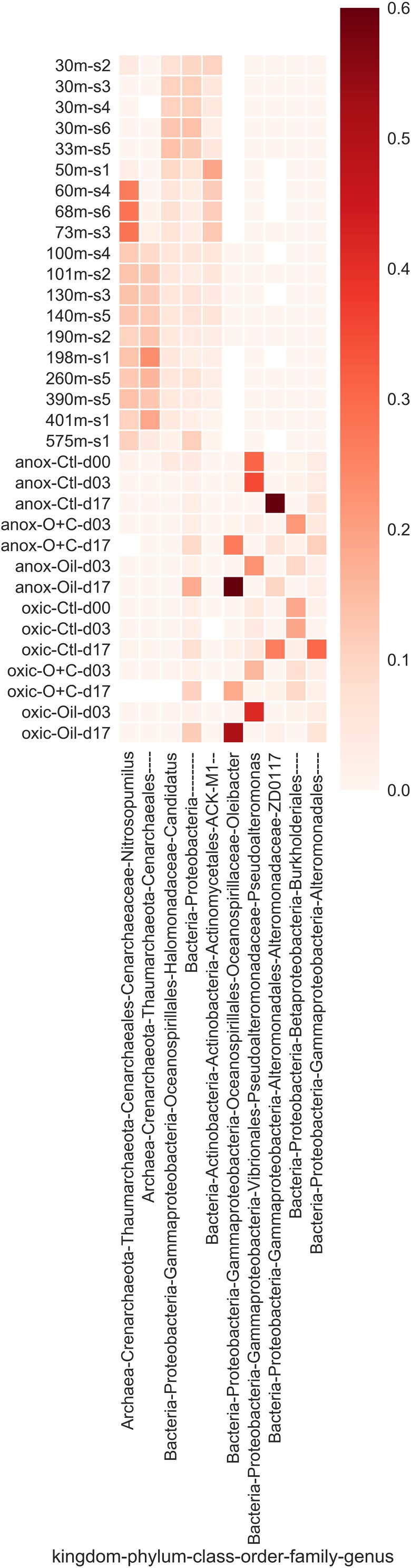
The most abundant microbial taxa that were enriched in either the natural or microcosm communities. Relative abundance of each taxon is represented by intensity of the heat map. Enriched taxa were determined using Fisher’s exact test.

There were important distinctions between the shallow and deep-water communities at all sample sites (Figure 4; Fisher’s exact test, *P* value < 0.05). The shallow waters were dominated by *Bacteria*, comprising 98.8% (±1.6%); *Archaea* comprised only ∼1% of reads in the shallow waters. The dominant phylum was *Proteobacteria* (38.4 ± 7.0% of reads) followed by *Bacteroidetes* (20.4 ± 2.0%), and *Cyanobacteria* (12.3 ± 3.9%). The most abundant Bacterial orders were *Flavobacteriales* (15.6 ± 1.4%), unclassified *Proteobacteria* (10.3 ± 2.2%), and MWH-UniP1 (2.2 ± 0.7%).

*Bacteria* also dominated the deep waters but declined from >98 to 70.8% (±7.6%) of all reads in deep water communities. *Archaea* increased in abundance compared to the shallow-water communities comprising 28.2% (±7.3%) of all reads in deep-water communities. The most abundant phylum was *Crenarchaeota* (26.4 ± 6.7%), followed closely by *Proteobacteria* (25.0 ± 9.9%). Other highly abundant Bacterial phyla included *Bacteroidetes* (9.7 ± 3.4%), *Chloroflexi* (4.7 ± 2.5%), and *Cyanobacteria* (3.4 ± 2.6%), although *Bacteroidetes* and *Cyanobacteria* declined to less than half of their relative abundance compared to shallow-waters.

The most abundant taxonomic was *Cenarchaeales* order (26.4 ± 6.7%), which comprised mostly of the *Archaea*. Following *Cenarchaeales*, the remaining highly abundant orders were all *Bacteria* and were more evenly distributed. These included *Flavobacteriales* (7.8 ± 2.9%), unclassified *Proteobacteria* (4.2 ± 2.3%), and *Phycisphaerales* (2.6 ± 1.3%).

**FIGURE 5:**
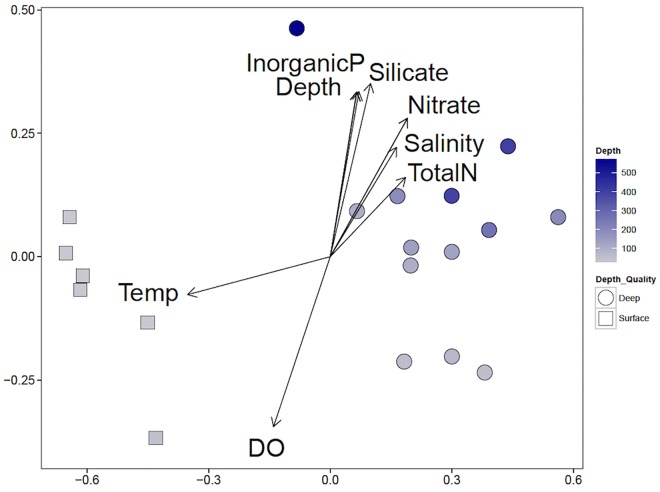
Non-metric multidimensional scaling (NMDS) correlation biplot of shallow and deep-water communities and influential environmental variables. NMDS of weighted Unifrac distances (stress 0.075, *P*-value <0.05) are shown. Shallow water communities are marked with squares, and deep-water communities are marked with circles. The blue intensity of the marker indicates the depth, with the shallowest samples in gray and the deepest samples in dark blue. Environmental variables were fit to the two-dimensional representation of the microbial communities; arrow directions indicate correlation with each axis and the size of the length of the arrow indicates the correlation coefficient.

Non-metric multidimensional scaling of weighted Unifrac distances between samples implies clusters of the communities according to depth (Figure 5). Temperature and DO were high in the surface waters and are influential factors on those communities (Table 2). Inorganic phosphate, silicate, nitrate, total nitrogen, and salinity increased with depth and are likely to be influential on the deep-water communities.

**Table 2:** Summary of environmental variables by depth category.

Environmental Parameter (^∗^*p* < 0.05)	Shallow (≤50 m, μ + σ^2^)	Deep (>50 m, μ + σ^2^)
Temperature^∗^	20.239 (±5.097)	7.240 (±1.362)
Dissolved oxygen (mg/L)^∗^	8.381 (±0.170)	4.278 (±2.069)
Salinity (PSU)^∗^	11.245 (±0.055)	11.327 (±0.042)
Nitrate (μmol/L)^∗^	0.152 (±0.053)	6.441 (±4.138)
Nitrite (μmol/L)	0.042 (±0.034)	0.028 (±0.010)
Ammonia (μmol/L)	0.024 (±0.010)	0.021 (±0.003)
Total nitrogen (mg/L)^∗^	0.397 (±0.033)	0.440 (±0.041)
Inorganic phosphate (μmol/L)^∗^	0.026 (±0.008)	0.606 (±0.569)
Silicate (μmol/L)^∗^	2.081 (±1.626)	36.450 (±23.317)
TOC (mg/L)	7.169 (±0.784)	6.820 (±0.463)

*Environmental variables for shallow (≤50 m) and deep (>50 m) waters were tested for statistically different means using a two-tailed t-test. Statistically significant environmental variables (P-value < 0.05) are indicated by ^∗^.*

#### Laboratory Microcosm Experiments

Water samples from the Caspian Sea were shipped to Tennessee to investigate oil hydrocarbon biodegradation in microcosm experiments. Microcosms were amended with oil (100 ppm) or oil (100 ppm) and Corexit (1 ppm). A control without oil and a killed control were also included (see section “Materials and Methods” for details). We used TEXMEX to analyze the changes in microbial community composition at day 17 (Tables 3, 4 and Supplementary Figures 2, 3). Briefly, TEXMEX transforms the OTU read counts using a Poisson lognormal distribution, enabling comparison of read abundance data across samples from different experimental conditions (e.g., control vs. oil-amended) and time points. *Oceanospirillaceae* (*Oleispira*) responded strongly to oil amendment in both oxic and anoxic microcosms. Three additional *Oceanospirillaceae* genera (*Amphritea*, *Neptunomonas*, *Oceanisperpentilla*) increased in abundance following oil amendment under oxic conditions but not under anoxic conditions. Overall, TEXMEX indicates that 31 genera responded positively to oil amendment in oxic conditions (Table 4) while only four genera did so in anoxic conditions (Table 3).

**Table 3:** List of taxa that were enriched in an anoxic, oil amended microcosm as determined by TEXMEX analysis.

kingdom	phylum	class	order	family	genus
*Bacteria*	*Bacteroidetes*	*Bacteroidia*	*Bacteroidales*	*Porphyromonadaceae*	*–*
*Bacteria*	*Proteobacteria*	*Gammaproteobacteria*	*Oceanospirillales*	*–*	*–*
*Bacteria*	*Proteobacteria*	*Gammaproteobacteria*	*Oceanospirillales*	*Oceanospirillaceae*	*–*
*Bacteria*	*Proteobacteria*	*Gammaproteobacteria*	*Oceanospirillales*	*Oceanospirillaceae*	*Oleispira*

*Changes in the microbial community composition of the anoxic, oil-amended microcosm were compared with that of the anoxic, non-amended microcosm. Microbial taxa that were enriched in the oil-amended microcosm relative to the non-amended microcosm are listed here.*

**Table 4:** List of taxa that were enriched in an oxic, oil amended microcosm as determined by TEXMEX analysis.

kingdom	phylum	class	order	family	genus
*Bacteria*	*Actinobacteria*	*Actinobacteria*	*Actinomycetales*	*Frankiaceae*	*–*
*Bacteria*	*Actinobacteria*	*Actinobacteria*	*Actinomycetales*	*Intrasporangiaceae*	*Phycicoccus*
*Bacteria*	*Actinobacteria*	*Actinobacteria*	*Actinomycetales*	*Intrasporangiaceae*	*Terracoccus*
*Bacteria*	*Bacteroidetes*	*Bacteroidia*	*Bacteroidales*	*–*	*–*
*Bacteria*	*Bacteroidetes*	*Cytophagia*	*Cytophagales*	*Cytophagaceae*	*Flectobacillus*
*Bacteria*	*Bacteroidetes*	*Flavobacteriia*	*Flavobacteriales*	*Flavobacteriaceae*	*Ulvibacter*
*Bacteria*	*Cyanobacteria*	*Nostocophycideae*	*Nostocales*	*–*	*–*
*Bacteria*	*Firmicutes*	*Clostridia*	*Clostridiales*	*Eubacteriaceae*	*Acetobacterium*
*Bacteria*	*Firmicutes*	*Clostridia*	*Clostridiales*	*Veillonellaceae*	*–*
*Bacteria*	*Proteobacteria*	*Alphaproteobacteria*	*Kiloniellales*	*–*	*–*
*Bacteria*	*Proteobacteria*	*Alphaproteobacteria*	*Kiloniellales*	*Kiloniellaceae*	*–*
*Bacteria*	*Proteobacteria*	*Alphaproteobacteria*	*Kordiimonadales*	*Kordiimonadaceae*	*–*
*Bacteria*	*Proteobacteria*	*Alphaproteobacteria*	*Rhizobiales*	*Bradyrhizobiaceae*	*Bosea*
*Bacteria*	*Proteobacteria*	*Alphaproteobacteria*	*Rhizobiales*	*Hyphomicrobiaceae*	*Parvibaculum*
*Bacteria*	*Proteobacteria*	*Alphaproteobacteria*	*Rhodobacterales*	*Hyphomonadaceae*	*Hyphomonas*
*Bacteria*	*Proteobacteria*	*Alphaproteobacteria*	*Rhodobacterales*	*Rhodobacteraceae*	*Phaeobacter*
*Bacteria*	*Proteobacteria*	*Alphaproteobacteria*	*Sphingomonadales*	*Erythrobacteraceae*	*Erythrobacter*
*Bacteria*	*Proteobacteria*	*Epsilonproteobacteria*	*–*	*–*	*–*
*Bacteria*	*Proteobacteria*	*Epsilonproteobacteria*	*Campylobacterales*	*–*	*–*
*Bacteria*	*Proteobacteria*	*Epsilonproteobacteria*	*Campylobacterales*	*Campylobacteraceae*	*–*
*Bacteria*	*Proteobacteria*	*Epsilonproteobacteria*	*Campylobacterales*	*Campylobacteraceae*	*Sulfurospirillum*
*Bacteria*	*Proteobacteria*	*Epsilonproteobacteria*	*Campylobacterales*	*Helicobacteraceae*	*Sulfuricurvum*
*Bacteria*	*Proteobacteria*	*Gammaproteobacteria*	*Alteromonadales*	*Alteromonadaceae*	*Glaciecola*
*Bacteria*	*Proteobacteria*	*Gammaproteobacteria*	*Alteromonadales*	*Alteromonadaceae*	*Simiduia*
*Bacteria*	*Proteobacteria*	*Gammaproteobacteria*	*Alteromonadales*	*Colwelliaceae*	*Thalassomonas*
*Bacteria*	*Proteobacteria*	*Gammaproteobacteria*	*Oceanospirillales*	*Oceanospirillaceae*	*–*
*Bacteria*	*Proteobacteria*	*Gammaproteobacteria*	*Oceanospirillales*	*Oceanospirillaceae*	*Amphritea*
*Bacteria*	*Proteobacteria*	*Gammaproteobacteria*	*Oceanospirillales*	*Oceanospirillaceae*	*Neptunomonas*
*Bacteria*	*Proteobacteria*	*Gammaproteobacteria*	*Oceanospirillales*	*Oceanospirillaceae*	*Oceaniserpentilla*
*Bacteria*	*Proteobacteria*	*Gammaproteobacteria*	*Oceanospirillales*	*Oceanospirillaceae*	*Oleispira*
*Bacteria*	*Proteobacteria*	*Gammaproteobacteria*	*Pseudomonadales*	*Pseudomonadaceae*	*–*

*Changes in the microbial community composition of the oxic, oil-amended microcosm were compared with that of the oxic, non-amended microcosm. Microbial taxa that were enriched in the oil-amended microcosm relative to the non-amended microcosm are listed here.*

**FIGURE 6:**
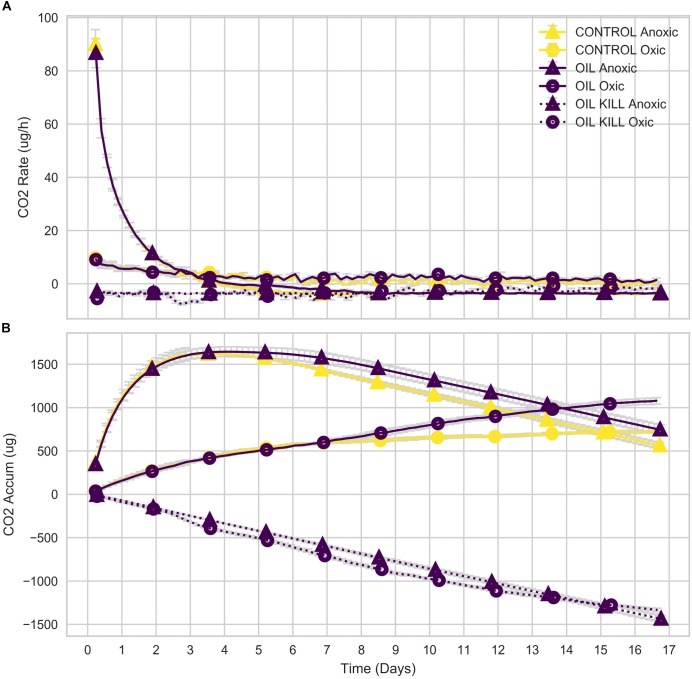
Respired CO_2_ of microcosms. Rate **(A)** and cumulative **(B)** CO_2_ generated by microbial communities in microcosm experiments.

Initially, the mean rate of CO_2_ generation in anoxic microcosms was high (∼90 μg/h), while that of oxic microcosms was low (∼10 μg/h; Figure 6A). The rate declined to 0 by day 8 in the anoxic microcosms but declined very slowly over the 17-day experiment in the oxic microcosms. The mean rate of CO_2_ generation in the oxic microcosms exceeded that of the anoxic microcosms by day 3, but, due to the initial high rate in the anoxic microcosms, cumulative CO_2_ generated by the oxic microcosms did not exceed that of anoxic microcosms until day 14 (oil amended) or day 15 (control; Figure 6B).

**FIGURE 7:**
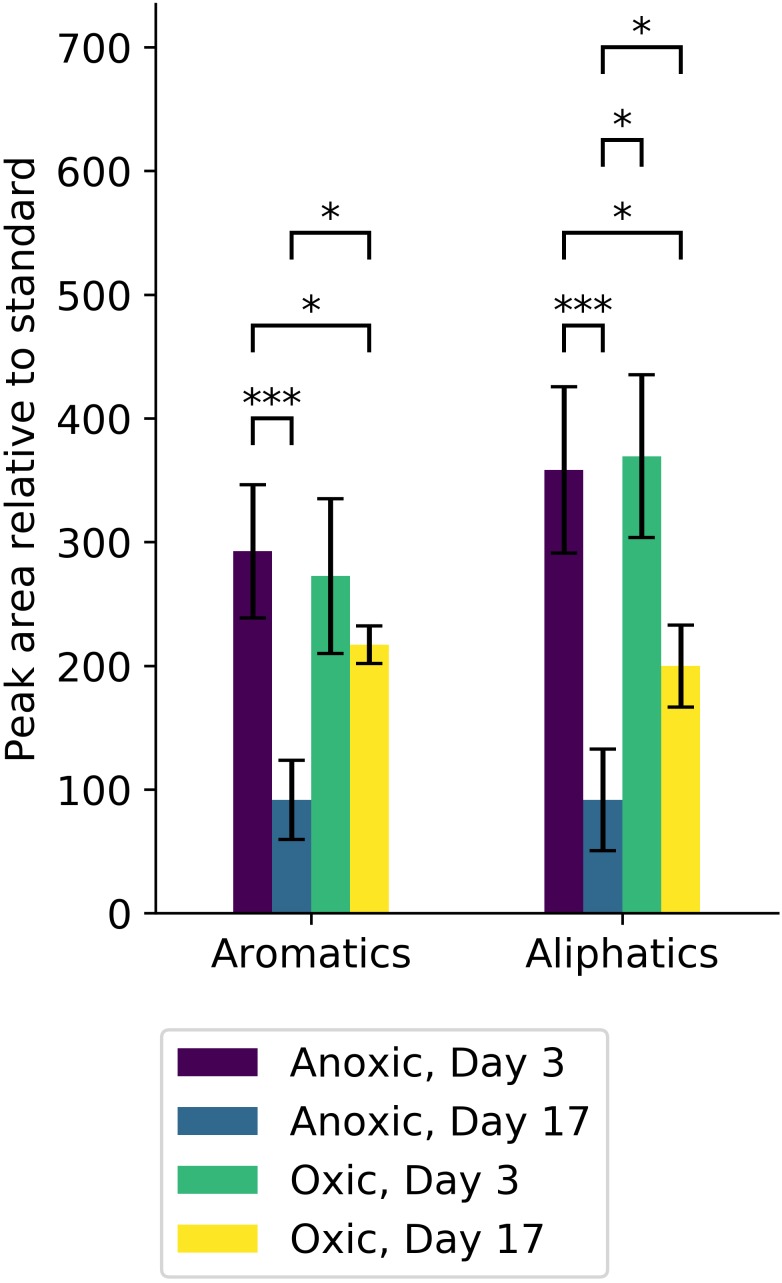
Hydrocarbons from microcosm experiments were quantified and are reported according to type (aromatic or aliphatic). Significant differences in the amount of each hydrocarbon type are indicated (^∗^alpha < 0.05, ^∗∗∗^alpha < 0.001). Anoxic microcosm communities (purple, blue) degraded significant amounts of both aromatic and aliphatic hydrocarbons, whereas oxic microcosm communities (green, yellow) did not. At day 17, the amount of both aromatic and aliphatic hydrocarbons was significantly less in anoxic microcosms (blue) compared to oxic microcosms (yellow).

Total aliphatic and aromatic hydrocarbons at days 3 and 17 were quantified relative to a standard (1,3-dichlorobenzene; Figure 7). At day 3, both aliphatic and aromatic hydrocarbon degradation were similar in oxic and anoxic conditions despite differences in cumulative CO_2_ respiration (Figure 6B). However, at day 17, anoxic microcosms degraded a larger fraction of total hydrocarbons compared to oxic, with a striking increase in aromatic hydrocarbon degradation.

**FIGURE 8:**
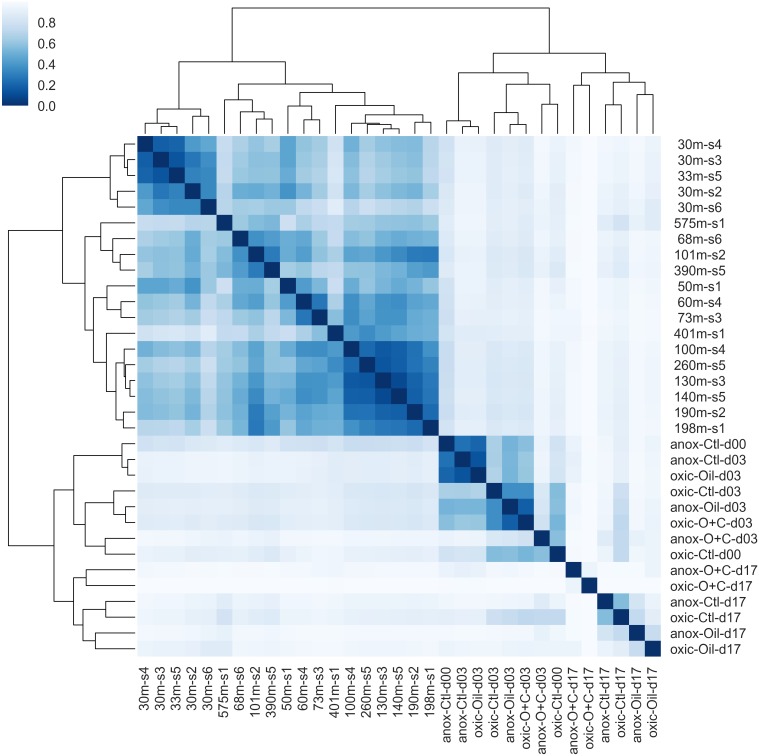
Hierarchical clustering of microbial communities. Bray-Curtis dissimilarity values between microbial communities were calculated and used for clustering. The dendrogram indicates the dissimilarity between clusters. The similarity between samples is indicated by intensity of the heatmap. Natural microbial communities clustered together and were distinct from microcosm communities. The shallow water communities (≤30 m) formed a sub-cluster within the natural communities. The early time points (days 0 and 3) formed a sub-cluster within the microcosm communities.

Degradation of aliphatics decreased inversely with size up to ∼22 carbons in both oxic and anoxic conditions. Anoxic microcosms degraded more of these aliphatic hydrocarbons. Aliphatic hydrocarbons of length 19 and 21 carbons were poorly degraded in oxic conditions relative to anoxic conditions. On the other hand, the heaviest aliphatics (>28) were more completely degraded in oxic microcosms compared to anoxic microcosms. Pristane, norpristane, and phytane were also more completely degraded in anoxic microcosms, with only a minor fraction degraded in oxic microcosms. Trimethyldodecane and trimethyltridecane were, similarly, lost from both oxic and anoxic conditions.

Aromatic hydrocarbons were degraded to a lesser extent than aliphatic hydrocarbons. This was particularly noticeable under oxic conditions. Both oxic and anoxic microcosms degraded similar proportions of 1,3-dimethylnapthalene, 1,6,7-trimethylapthalene, 1,6-dimethylnapthalene, 1-methylnaphthalene, 2,6-dimethylnaphthalene, 2-methylnaphthalene, biphenyl, and benzothiophenes.

The EPA lists sixteen hazardous hydrocarbons (collectively “EPA16”). Of these EPA16 hydrocarbons, oxic microcosms degraded naphthalene more than any other (Supplementary Figure 4). More of the EPA16 hydrocarbons were degraded in anoxic microcosms. In particular, benz(a)anthracene and chrysene were completely depleted in anoxic microcosm experiments. Naphthalene was also depleted, similarly, to oxic microcosms. Oxic microcosms degraded phenanthrene more efficiently than anoxic microcosms. In addition to the EPA16 compounds already mentioned, higher proportions of dibenzothiophene were degraded in anoxic microcosms.

## Discussion

Eutrophication in the Caspian Sea has caused the deep waters to become severely hypoxic (Figure 2 and Table 2). Although this environment has a long history of natural exposure to oil hydrocarbons, the concentration of oil hydrocarbons has increased due to increased oil exploration in the recent past. Microbial communities degrade oil under both aerobic and anaerobic conditions (Coates et al., 1996). The purpose of this study was to characterize the *in situ* community structure, and then to investigate the ability of that community to degrade oil hydrocarbon under oxic or anoxic conditions. Baseline sampling provides a snapshot of the native, ambient community structure on the date of sampling, representing non-perturbed environmental conditions, which is an important reference for experimental studies. We used microcosm experiments to investigate changes in the microbial community composition and the potential of this community for oil hydrocarbon biodegradation under oxic and anoxic conditions. We report here the changes in community composition and evaluate those changes with respect to the baseline community. We also report the extent of degradation of various oil hydrocarbons and the differences in degradation.

As expected, the shallow (≤50 m) and deep (>50 m) water communities of the Caspian were different (Figure 4, 6). NMDS indicates that communities tend to cluster by depth (Figure 4). Several environmental variables were important in distinguishing the communities. Temperature and DO were inversely correlated with the depth of the community, while inorganic phosphate, silicate, nitrate, salinity, and total nitrogen were positively correlated with depth of community. The most abundant phyla were *Proteobacteria* (30% of all reads), *Crenarchaeota* (18%), and *Bacteroidetes* (13%) (Figure 3). *Proteobacteria* dominated the shallow water communities (38%) but comprised a substantial fraction at all sites and depths. *Proteobacteria* may be enriched during oil spills in other regions of the world (Grossman et al., 2000; Hazen et al., 2010; Bælum et al., 2012). *Bacteroidetes* were observed in highest abundance in the shallow water communities (20%) and declined with depth. *Bacteroidetes* have been observed as co-dominant in oil-impacted seawater (Kasai et al., 2002). Our results indicate that *Proteobacteria* and *Bacteroidetes* are dominant phyla in the Caspian Sea, which is consistent with reports of these phyla in other oil impacted basins and with reports that the Caspian Sea receives large inputs of petroleum hydrocarbons (Chicherina et al., 2004). *Crenarchaeota* dominated most of the deep-water communities (26%), suggesting that these communities may be nutrient limited since this group predominantly survives by ammonia-oxidation (Hazen et al., 2016).

*Flavobacteriales*, *Actinomycetales*, *and Oceanospirillales* orders co-dominated the ambient communities (Figure 3). *Flavobacteriales* have previously been reported as dominant in shallow water communities (Liu et al., 2017), and members of this order are commonly reported as chemoheterotrophs and hydrocarbon degraders (Abbasian et al., 2015a). In oxic microcosms, *Actinomycetales* and *Oceanospirillales* orders were enriched with oil-amendment (Tables 3, 4 and Supplementary Figures 2, 3). *Gammaproteobacteria* are commonly found in oil-impacted marine environments and dominate oil amended microcosm experiments for ≥20 days (Röling et al., 2002; Hazen et al., 2016); their (co)dominance therefore suggests that these communities are capable of oil biodegradation. Interestingly, unclassified *Proteobacteria* comprised a substantial fraction of the shallow water communities (Figure 3 and Supplementary Figure 6). It is unknown what role these unclassified microbes may play, if any, in hydrocarbon degradation.

Deep-water communities were dominated by *Cenarchaeales* (Figure 3 and Supplementary Figure 6). *Thaumarchaeota*, known ammonia-oxidizing *Archaea*, are thought to play an important role in nitrogen cycling and may be important in the deep waters of the Caspian Sea (Mahmoudi et al., 2015; Techtmann et al., 2017). They have previously been reported to dominate the communities in the Baltic Sea, which, like the Caspian Sea, is characterized by low salinity and low oxygen concentration (Labrenz et al., 2010). It has been hypothesized that these *Thaumarchaeota* are adapted to eutrophic conditions and are not obligate autotrophic ammonia oxidizers (Techtmann et al., 2017). *Cenarchaeales* have been observed in polychlorinated biphenyl and PAH contaminated sand samples (Harada et al., 2013) and contribute to the degradation of biphenyl in activated biosludge (Song et al., 2018). Although *Bacteria* dominated all samples (at all depths), they decreased in abundance with depth, while *Archaea* increased in abundance with depth. *Planctomycetes*, however, were enriched in the deep waters in contrast with other *Bacterial* phyla. *Phycisphaerales* were commonly observed in the deep-water communities (2%), which was unexpected because they are known to be enriched in aerobic seawater (Wang et al., 2018). Curiously, although *Gammaproteobacteria* tended to decline with depth (consistent with *Bacteria* in general), the highest fraction was observed in the deepest sample (36%, site 1). Two-thirds of these *Gammaproteobacteria* were unclassified at the order level, and these unclassified reads dominated the community (23%). Other *Bacteria* that increased in abundance uniquely in this sample included SAR406 AB16 Arctic96B-7 (3% compared to <1% in general) and WS3 PRR-12 GN03 (∼1% of reads but rarely observed otherwise).

Opportunistic organisms capable of metabolizing oil hydrocarbons may be ubiquitous globally in deep sea basins, but they vary in both biodegradation capabilities and proportional contribution to the community structure (Hazen et al., 2016). Population blooms of these hydrocarbon degrading *Bacteria* are expected with an influx of oil hydrocarbons into a system that is likely to occur from increased oil exploration and recovery. Additionally, the deep waters of the Caspian Sea are known to be severely hypoxic. Due to the unique combination of exposure history to oil hydrocarbons and persistent hypoxia, the deep waters of the Caspian may harbor communities with novel adaptations for anoxic hydrocarbon degradation. Therefore, we investigated the oil hydrocarbon biodegradation potential of this community under oxic and anoxic conditions.

TEXMEX highlights organisms that increased in abundance in oil-amended microcosms compared to organisms that increased in both control and oil-amended microcosms, thus mitigating the impact of so-called “bottle effects” (Olesen et al., 2016) (Tables 3, 4 and Supplementary Figures 2, 3). TEXMEX revealed that *Oceanospirillales*, which includes known oil degraders (Hazen et al., 2016), responded to oil amendment in both oxic and anoxic microcosm experiments. However, at least three genera were enriched under oxic conditions that were not enriched under anoxic conditions. The *Oceanospirillales* that responded in both oxic and anoxic microcosms may be facultative anaerobes or aerotolerant, while those responding only in oxic conditions may be facultative anaerobes or obligate aerobes. In anoxic microcosms, the *Sphingomonadales* order responded strongly to oil amendment, which is consistent with reports that members of this order are hydrocarbon degraders (Beazley et al., 2012). *Sphingomonadales* are found in aerobic waters (Abbasian et al., 2015b), however, those identified in this study were unclassified below the order level and may represent novel taxa capable of hydrocarbon degradation in anoxic environments.

*Alphaproteobacteria* also responded to oil amendment in oxic microcosms (Table 4). Two *Rhodobacterales* genera responded to oil amendment, which is consistent with previous reports (Chakraborty et al., 2012). *Rhodobacterales* (*Phaeobacter*) are commonly reported to be marine heterotrophs capable of growing on a wide range of nitrogen and phosphorous concentrations and in a wide range of temperatures (Lee et al., 2016; Trautwein et al., 2017; Wienhausen et al., 2017). Unclassified *Kiloniellales* (*Kiloniellaceae*) also responded to oil amendment, and, although many are host associated (Soffer et al., 2015; Cleary et al., 2016; Lawler et al., 2016), one member of this order was isolated and reported as a chemoheterotrophic aerobe that may be involved in denitrification (Wiese et al., 2009).

Oxic and anoxic microcosms degraded similar amounts of hydrocarbons by day 3, but there were important differences in hydrocarbon degradation by day 17 (Figure 7, 8) that may be attributed to changes in microbial community composition during that period. Interestingly, anoxic microcosms degraded more total oil hydrocarbons by day 17 compared to oxic microcosms. The half-life for total hydrocarbon degradation was estimated to be 11 days in anoxic microcosms and 15 days in oxic microcosms. Most of the individual oil hydrocarbons were degraded more efficiently in anoxic microcosms (Supplementary Figures 4, 5). This is especially curious in light of the fact that CO_2_ respiration in the anoxic microcosms ceased by day 8. Anoxic microcosms degraded a larger proportion of the shorter aliphatics (<22 carbons; including branched aliphatics, e.g., pristane and phytane) than oxic microcosms, while oxic degradation of longer aliphatics (≥22 carbons) exceeded anoxic degradation (Supplementary Figures 4, 5). Anoxic degradation of all aromatic hydrocarbons was comparable to or exceeded oxic degradation. In particular, degradation of benz(a)anthracene, chrysene, dibenzothiophene, fluorene, and phenanthrene in anoxic microcosms far exceeded that of oxic microcosms. These results suggest that deep water microbial communities in the Caspian Sea are adapted for better oil biodegradation under anoxic and (*in situ* hypoxic) conditions when compared to oxic conditions. Potential oil degradation pathways have been identified in *Gammaproteobacteria*, including pathways for the degradation of low molecular weight alkanes, aromatics, BTEX, proline, catechol, cyclohexanone, and nitroalkanes (Hu et al., 2017). It is therefore reasonable to expect that the *Gammaproteobacteria* observed in these results are important in the biodegradation results observed here, which is consistent with other reports (Bælum et al., 2012).

Phytane, pristane, and hopane are used as analytic standards when attempting to determine the origin of crude oil and its rate of biodegradation. Pristane and phytane are common components of crude oil, and the pristane/phytane ratio may be used as an indicator of the oil source (Rashid, 1979; Seklemova et al., 2001). However, pristane and phytane are known to be susceptible to biodegradation (Guo et al., 2010), which was also observed in this study. Hopane is considered less susceptible to biodegradation than phytane and pristane (Gagni and Cam, 2007; Guo et al., 2010); however, some biodegradation of hopane was also observed in this study. These findings have important implications for the analysis of crude oil biodegradation in the Caspian Sea and elsewhere.

The deep waters of the Caspian Sea are both hypoxic and cold (6°C) and may be nutrient limited. Hydrocarbons may be degraded more efficiently at low temperatures compared to warmer temperatures, which could explain high hydrocarbon degradation in the deep waters of the Caspian. However, improved hydrocarbon degradation under anoxic (compared to oxic) atmospheric headspace, as observed and reported here, is not well understood. The changes in microbial community composition likely account for the observed changes in oil hydrocarbon biodegradation. It is also possible that these microorganisms have adapted to the unique conditions of the deep-waters of the Caspian Sea with novel mechanisms to degrade recalcitrant hydrocarbons that may be inhibited under anoxic conditions; future meta’omics studies will be required to determine if this is the case. Furthermore, future work should include microcosm experiments with more varied nutrient conditions. Many of the reads observed in the microcosm communities were not able to be classified, even at high taxonomic levels. These reads represent potentially novel organisms that may be amenable to growth in laboratory conditions, either in isolation or as a consortium, and should be investigated further. Based on the enrichments observed in this study, many of these unclassified taxa may be oil degraders. Deep metagenomic and metatranscriptomic sequencing of both microcosm experiments and ambient communities should be combined with isolation of microorganisms and characterization of their phenotypes to better understand the mechanism of rapid anoxic oil-biodegradation observed in this study.

## Author Contributions

JM, TH, NM, and ST wrote the manuscript and provided analysis. SO and EA conducted the Bioinformatics. AF, PG, JF, and JL conducted the lab analysis. ST, JF, DJ, NG, and FA conducted field sampling.

## Conflict of Interest Statement

The authors declare that the research was conducted in the absence of any commercial or financial relationships that could be construed as a potential conflict of interest.
