# Supplementary material for: Oil Hydrocarbon Degradation by Caspian Sea Microbial Communities
Source: Front Microbiol. 2019 May 9;10:995. doi: 10.3389/fmicb.2019.00995 (PMC6521576; doi:10.3389/fmicb.2019.00995)
Supplement: Supplementary file 1 [file Data_Sheet_1.PDF]

# 1 Supplemental Tables

Supplemental Table 1. Sample Locations.

| Station ID | Sample Depth (m) | Latitude | Longitude |
|---|---|---|---|
| **1** | 50 | 39.745591 | 50.4806 |
| | 198 | 39.745591 | 50.4806 |
| | 401 | 39.745591 | 50.4806 |
| | 575 | 39.745591 | 50.4806 |
| **2** | 30 | 39.990257 | 51.500808 |
| | 101 | 39.990257 | 51.500808 |
| | 190 | 39.990257 | 51.500808 |
| **3** | 30 | 40.040589 | 51.347301 |
| | 73 | 40.040589 | 51.347301 |
| | 130 | 40.040589 | 51.347301 |
| **4** | 30 | 40.07526 | 51.287336 |
| | 60 | 40.07526 | 51.287336 |
| | 100 | 40.07526 | 51.287336 |
| **5** | 33 | 40.019905 | 51.264487 |
| | 140 | 40.019905 | 51.264487 |
| | 260 | 40.019905 | 51.264487 |
| | 390 | 40.019905 | 51.264487 |
| **6** | 30 | 40.284338 | 50.932697 |
| | 58 | 40.284338 | 50.932697 |

Supplemental Table 2. Redfield ratios.

| Site | Depth | N:P (ratio) |
|---|---|---|
| 1 | 50 | 8.78 |
| | 198 | 14.89 |
| | 401 | 8.17 |
| | 575 | 2.32 |
| 2 | 30 | 7.30 |
| | 101 | 19.74 |
| | 190 | 14.68 |
| 3 | 30 | 7.68 |
| | 73 | 25.46 |
| | 130 | 16.46 |
| 4 | 30 | 4.33 |
| | 60 | 65.17 |
| | 100 | 21.85 |
| 5 | 33 | 7.62 |
| | 140 | 20.76 |
| | 260 | 14.61 |
| | 390 | 9.00 |
| 6 | 30 | 8.41 |
| | 58 | 16.43 |

Supplemental Table 3. Alpha-diversity and richness of natural communities from environmental samples. The number of reads (n Reads) and number of OTUs (n OTUs) were counted directly. Alpha-diversity for each sample was calculated with the Shannon and Simpson metrics. Shannon diversity index is more sensitive to rare OTUs, while the Simpson diversity index is more sensitive to dominant OTUs. The chao1 metric was used to estimate the actual richness for each sample.

| Sample ID | n Reads | n OTUs | Shannon | Simpson | chao1 |
|---|---|---|---|---|---|
| **30m-s2** | 113056 | 273 | 5.32 | 0.956 | 328.12 |
| **30m-s3** | 67118 | 211 | 5.08 | 0.946 | 250.00 |
| **30m-s4** | 47754 | 201 | 5.20 | 0.953 | 261.00 |
| **30m-s6** | 126582 | 345 | 5.09 | 0.945 | 452.50 |
| **33m-s5** | 65308 | 212 | 5.01 | 0.943 | 253.40 |
| **50m-s1** | 66159 | 228 | 4.91 | 0.930 | 250.96 |
| **60m-s4** | 58698 | 249 | 4.75 | 0.901 | 315.30 |
| **68m-s6** | 155979 | 479 | 4.88 | 0.895 | 528.22 |
| **73m-s3** | 36320 | 244 | 4.74 | 0.893 | 284.28 |
| **100m-s4** | 53599 | 334 | 5.56 | 0.956 | 380.24 |
| **101m-s2** | 139458 | 367 | 5.47 | 0.949 | 420.32 |
| **130m-s3** | 63159 | 413 | 5.59 | 0.951 | 463.62 |
| **140m-s5** | 71018 | 370 | 5.49 | 0.951 | 423.63 |
| **190m-s2** | 84278 | 508 | 5.90 | 0.959 | 565.50 |
| **198m-s1** | 88564 | 311 | 5.00 | 0.916 | 346.65 |
| **260m-s5** | 55545 | 455 | 5.51 | 0.944 | 519.48 |
| **390m-s5** | 206461 | 598 | 5.74 | 0.951 | 642.68 |
| **401m-s1** | 31103 | 292 | 5.35 | 0.940 | 327.25 |
| **575m-s1** | 184878 | 464 | 4.94 | 0.913 | 524.02 |

Supplemental Table 4. Alpha-diversity and richness of microcosm communities. The number of reads (n Reads) and number of OTUs (n OTUs) were counted directly. Alpha-diversity for each sample was calculated with the Shannon and Simpson metrics. Shannon diversity index is more sensitive to rare OTUs, while the Simpson diversity index is more sensitive to dominant OTUs. The chao1 metric was used to estimate the actual richness for each sample.

| **Microcosm ID** | **n Reads** | **n OTUs** | **Shannon** | **Simpson** | **chao1** |
|---|---|---|---|---|---|
| **anox-Ctl-d00** | 53532 | 581 | 5.87 | 0.901 | 608.51 |
| **anox-Ctl-d03** | 38853 | 569 | 5.67 | 0.874 | 601.28 |
| **anox-Ctl-d17** | 1112199 | 593 | 2.07 | 0.496 | 630.01 |
| **anox-O+C-d03** | 1390713 | 816 | 5.07 | 0.919 | 822.90 |
| **anox-O+C-d17** | 5843 | 104 | 2.82 | 0.766 | 136.50 |
| **anox-Oil-d03** | 125942 | 651 | 6.03 | 0.934 | 667.02 |
| **anox-Oil-d17** | 957277 | 458 | 2.16 | 0.601 | 501.67 |
| **oxic-Ctl-d00** | 401172 | 753 | 5.54 | 0.937 | 784.92 |
| **oxic-Ctl-d03** | 113905 | 662 | 5.90 | 0.940 | 679.35 |
| **oxic-Ctl-d17** | 862007 | 764 | 3.97 | 0.828 | 796.22 |
| **oxic-O+C-d03** | 162166 | 763 | 6.63 | 0.962 | 789.05 |
| **oxic-O+C-d17** | 218 | 50 | 4.43 | 0.917 | 83.33 |
| **oxic-Oil-d03** | 40135 | 556 | 5.29 | 0.828 | 574.71 |
| **oxic-Oil-d17** | 188931 | 281 | 2.02 | 0.652 | 326.11 |

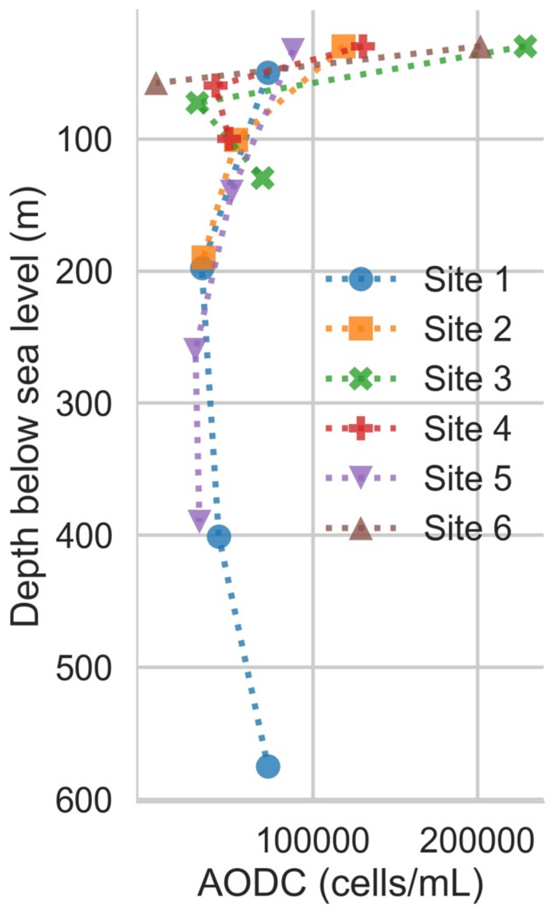


Supplemental Figure 1. Acridine orange direct counts (AODC).

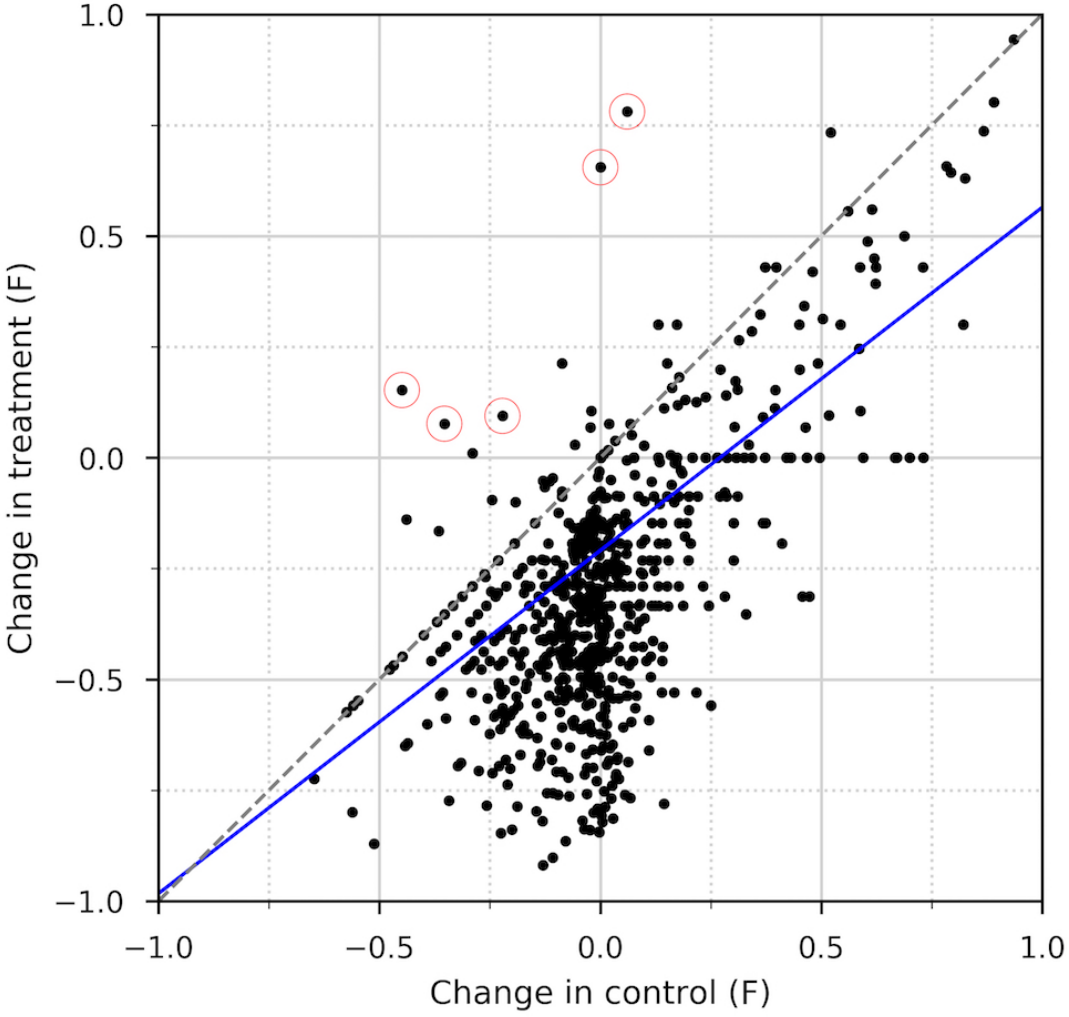


Supplemental Figure 2. Effect of oil amendment on anoxic microcosm communities. TEXMEX illustrates the taxa that responded strongly to oil amendment (vertical axis) in red circles compared to control (horizontal axis). The blue line is the linear best fit for the correlation between oil amended and control microcosms. The gray line represents no difference in the two conditions. Taxa that responded are listed in Table 3.

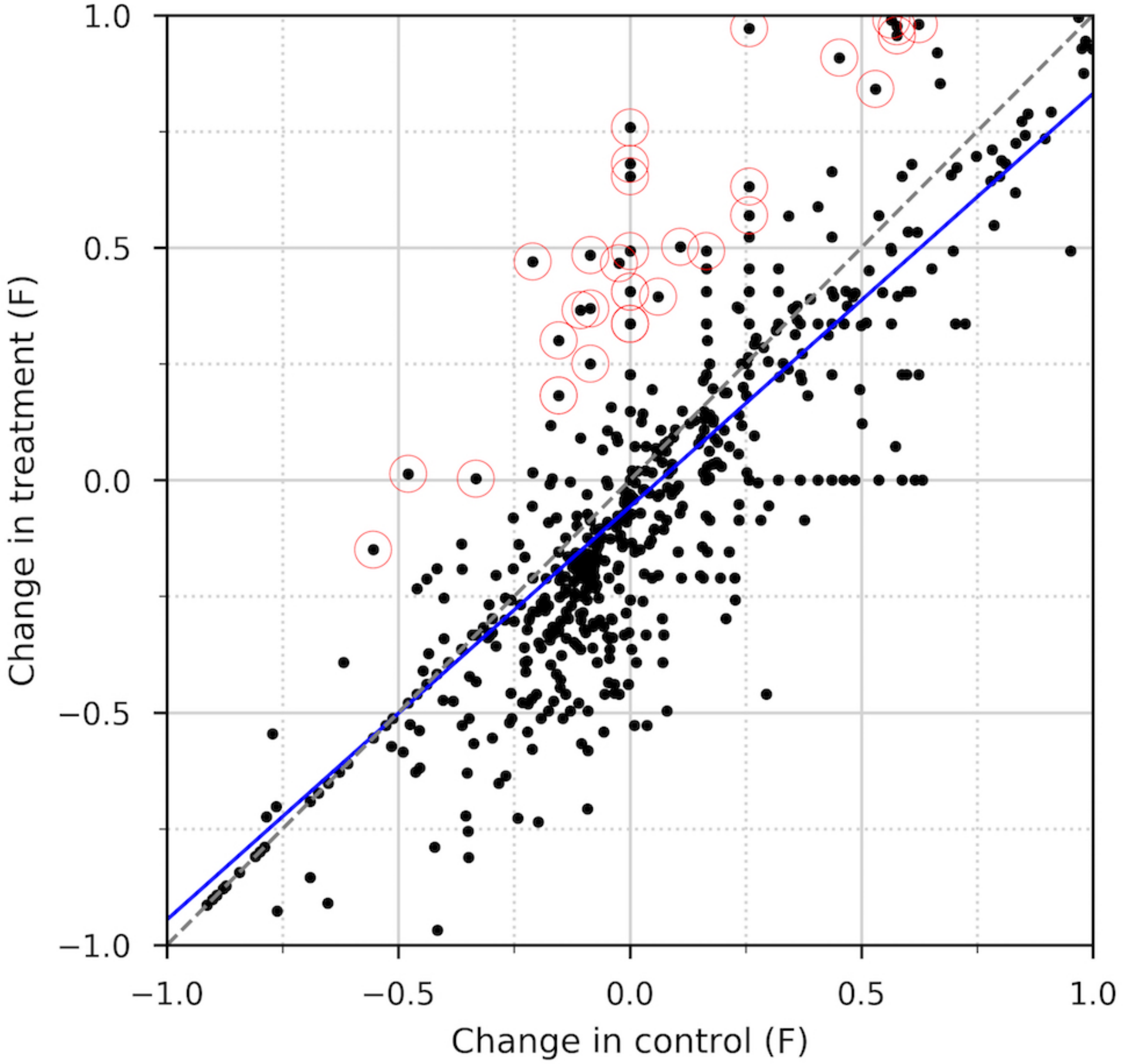


Supplemental Figure 3. Effect of oil amendment on oxic microcosm communities. TEXMEX illustrates the taxa that responded strongly to oil amendment (vertical axis) in red circles compared to control (horizontal axis). The blue line is the linear best fit for the correlation between oil amended and control microcosms. The gray line represents no difference in the two conditions. Taxa that responded are listed in Table 4.

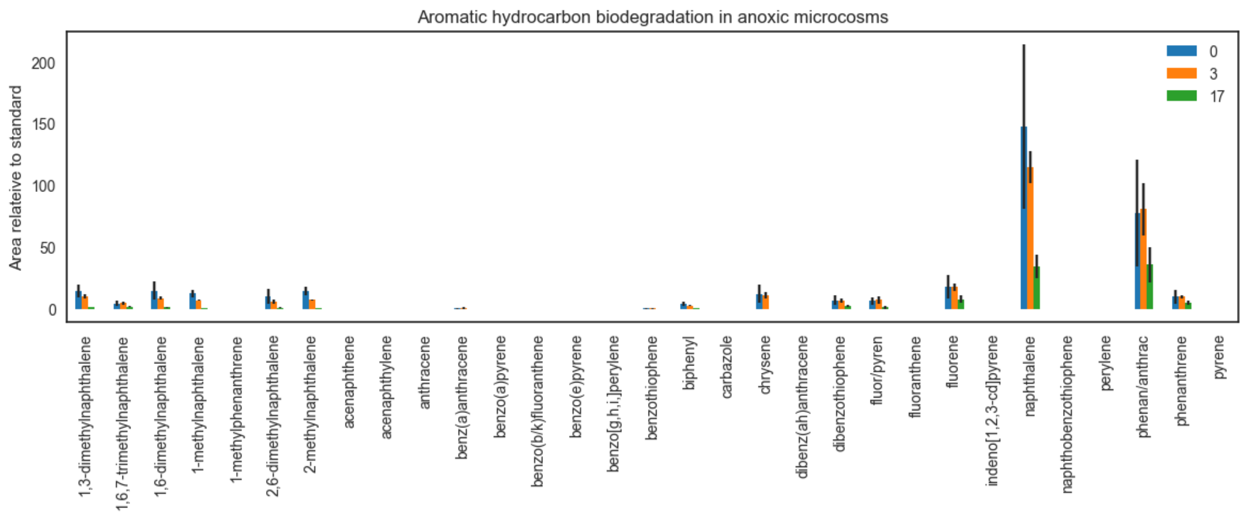


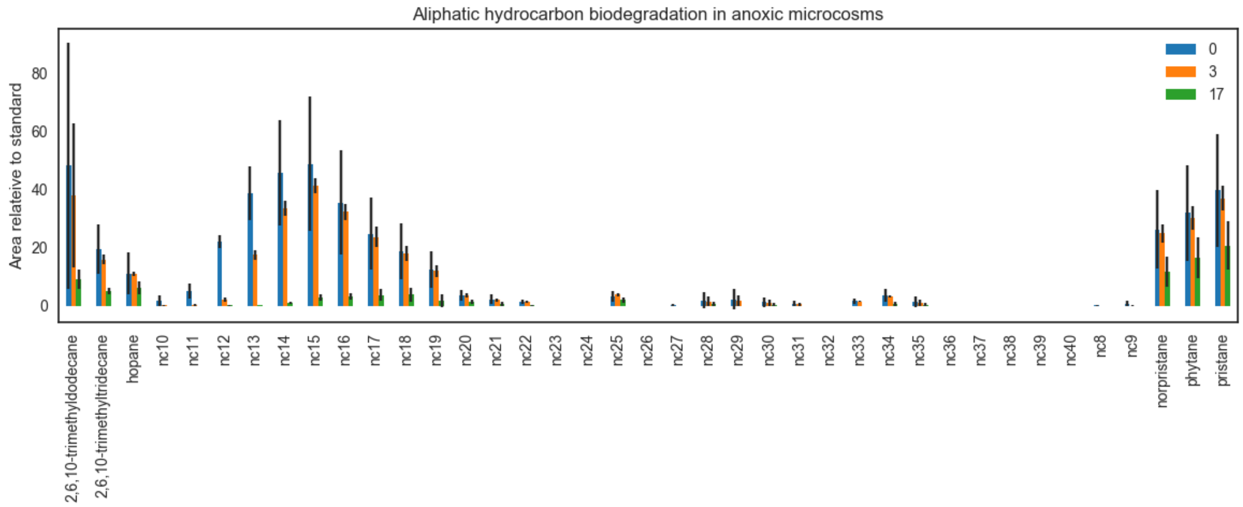


Supplemental Figure 4. Quantification of individual hydrocarbons in anoxic microcosm experiments.

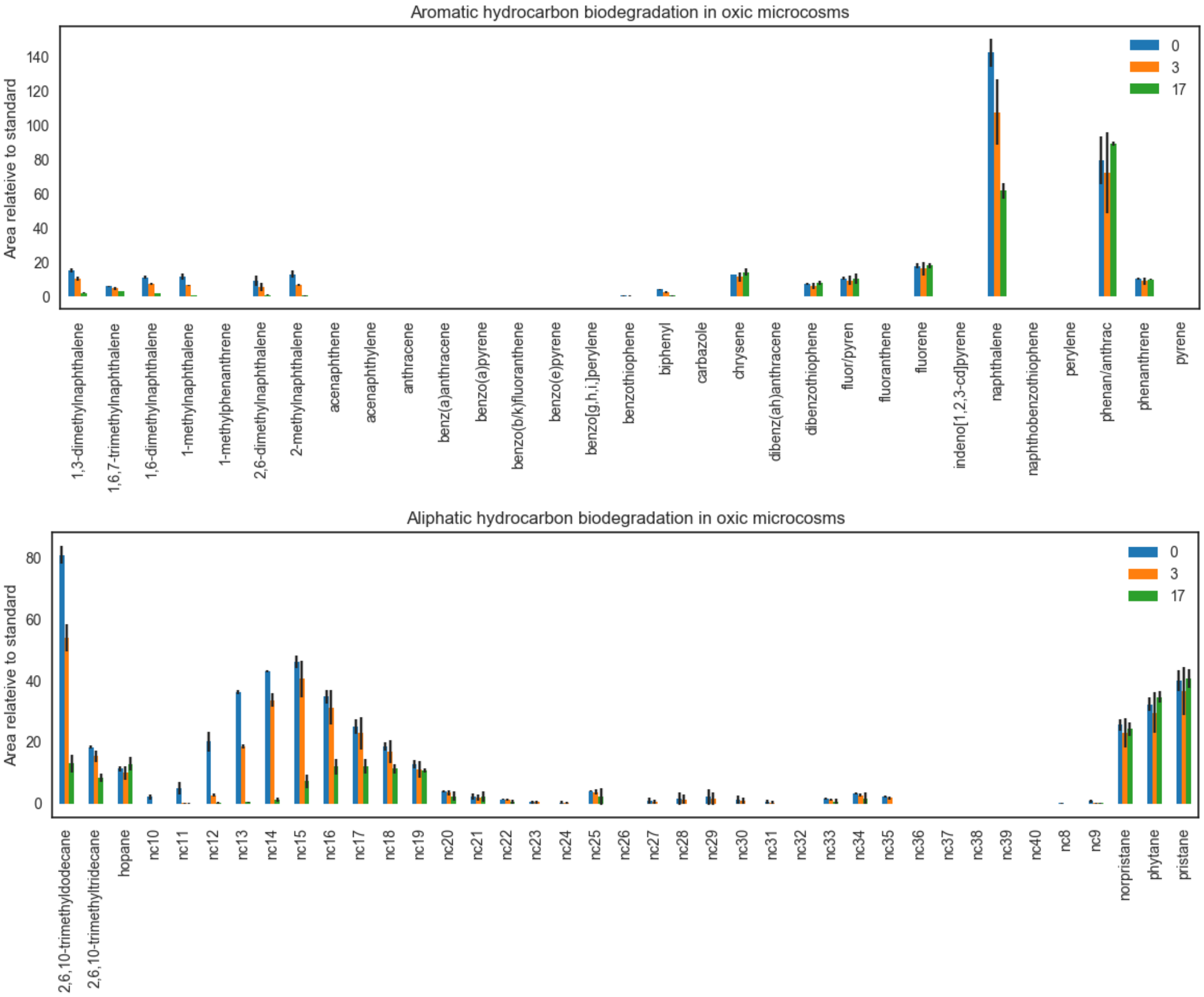


Supplemental Figure 5. Quantification of individual hydrocarbons in oxic microcosm experiments.

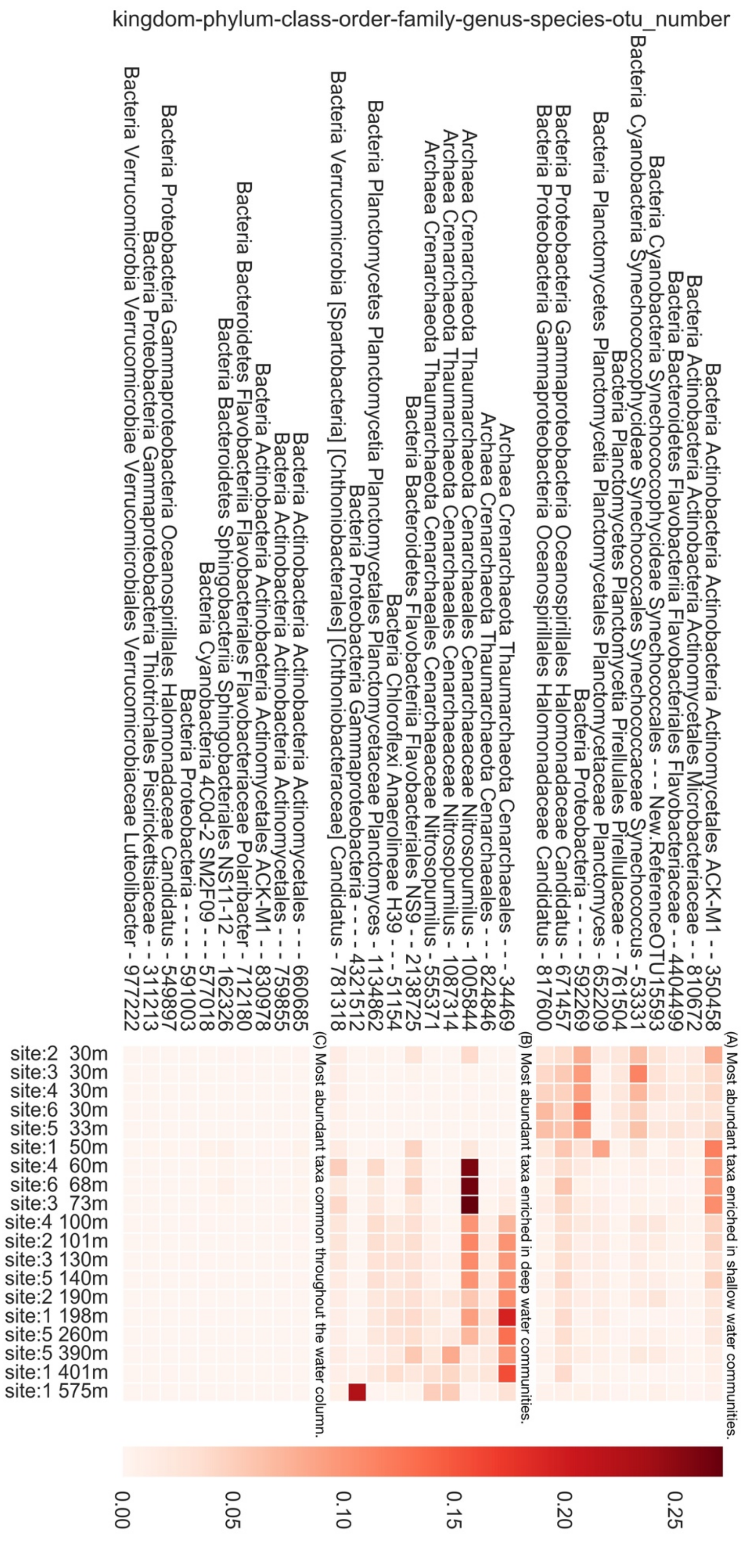


Supplemental Figure 6. Enriched taxa in shallow and deep-water communities. (A) Most abundant taxa that were enriched in the shallow water (≤50 m) communities. (B) Most abundant taxa that were enriched in the deep-water (>50 m) communities. (C) Most abundant that were common throughout the water column (not enriched in shallow or deep-water communities).
